# Supplementary figures and images for: Postnatal Development of Dendritic Morphology and Action Potential Shape in Rat Substantia Nigra Dopaminergic Neurons
Source: eNeuro. 2025 Apr 18;12(4):ENEURO.0413-24.2025. doi: 10.1523/ENEURO.0413-24.2025 (PMC12039477; doi:10.1523/ENEURO.0413-24.2025)

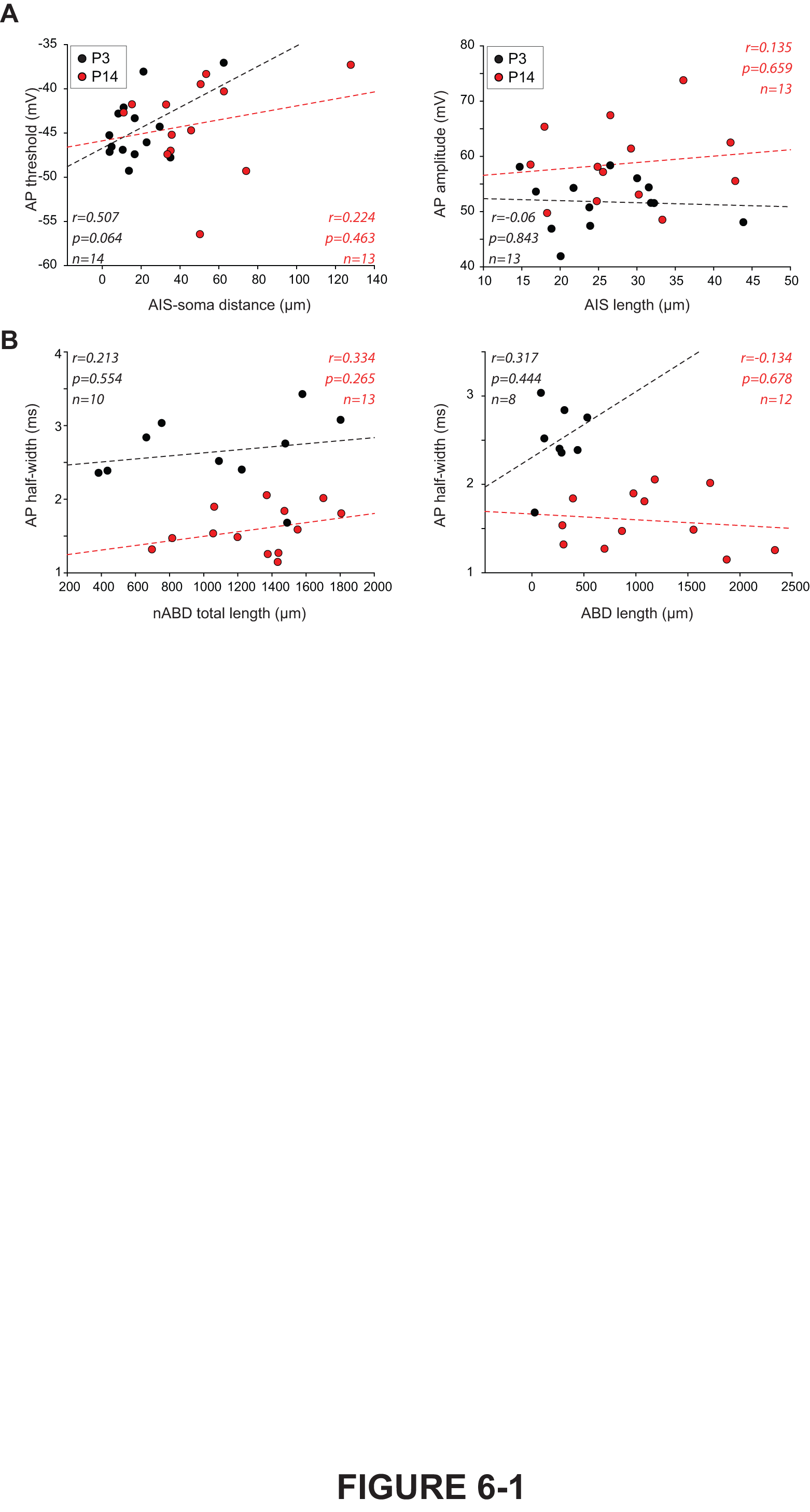

Supplement: Figure 6-1 — Relationships between morphology and AP shape in experimental data at P3 and P14. A, scatter plots showing the relationship between AIS-soma distance and AP threshold (left) and between AIS length and AP amplitude (right). B, scatter plots showing the relationship between nABD length (left) or ABD length (right) and AP half-width for P3 and P14 neurons. Dotted lines represent non-significant correlations. The r, p, and n values corresponding to the statistically significant linear regressions are presented on each scatter plot. Download Figure 6-1, TIF file. [file eneuro-12-ENEURO.0413-24.2025-s001.tif]
